# A Comparison of Two DNA Metagenomic Bioinformatic Pipelines While Evaluating the Microbial Diversity in Feces of Tanzanian Small Holder Dairy Cattle

**DOI:** 10.1155/2020/2348560

**Published:** 2020-04-22

**Authors:** Felix M. Kibegwa, Rawlynce C. Bett, Charles K. Gachuiri, Francesca Stomeo, Fidalis D. Mujibi

**Affiliations:** ^1^Department of Animal Production, University of Nairobi, Kenya; ^2^Biosciences eastern and central Africa-International Livestock Research Institute (BecA-ILRI) Hub, Nairobi, Kenya; ^3^USOMI Limited, Genomics Division, Nairobi, Kenya

## Abstract

Analysis of shotgun metagenomic data generated from next generation sequencing platforms can be done through a variety of bioinformatic pipelines. These pipelines employ different sets of sophisticated bioinformatics algorithms which may affect the results of this analysis. In this study, we compared two commonly used pipelines for shotgun metagenomic analysis: MG-RAST and Kraken 2, in terms of taxonomic classification, diversity analysis, and usability using their primarily default parameters. Overall, the two pipelines detected similar abundance distributions in the three most abundant taxa Proteobacteria, Firmicutes, and Bacteroidetes. Within bacterial domain, 497 genera were identified by both pipelines, while an additional 694 and 98 genera were solely identified by Kraken 2 and MG-RAST, respectively. 933 species were detected by the two algorithms. Kraken 2 solely detected 3550 species, while MG-RAST identified 557 species uniquely. For archaea, Kraken 2 generated 105 and 236 genera and species, respectively, while MG-RAST detected 60 genera and 88 species. 54 genera and 72 species were commonly detected by the two methods. Kraken 2 had a quicker analysis time (~4 hours) while MG-RAST took approximately 2 days per sample. This study revealed that Kraken 2 and MG-RAST generate comparable results and that a reliable high-level overview of sample is generated irrespective of the pipeline selected. However, Kraken 2 generated a more accurate taxonomic identification given the higher number of “Unclassified” reads in MG-RAST. The observed variations at the genus level show that a main restriction is using different databases for classification of the metagenomic data. The results of this research indicate that a more inclusive and representative classification of microbiomes may be achieved through creation of the combined pipelines.

## 1. Introduction

Metagenomics is a high-throughput sequencing (HTS) technique commonly used to investigate complex microbial communities in terms of composition, structure, diversity, and function. This culture-independent application has gained importance in microbiological studies over the past decade [1] especially in studies of environmental communities [2, 3], in industrial quality control processes [4], and in understanding the influence of gastrointestinal microbes on the health of human beings and their well-being [5]. The phrase metagenomics can be defined by two distinct approaches: targeted and shotgun metagenomics. Targeted metagenomics is also called amplicon-based metagenomics or metagenetics [6]. This technique focusses exclusively on a genomic marker, which is amplified before sequencing thus greatly reducing the amount of data to be sequenced and analyzed. Shotgun metagenomics on the other hand uses extraction and sequencing of the complete DNA to study the genomic content of a sample. Consequently, this integrated strategy provides a rich image of the microbiota and offers the chance to study the taxonomic classification and functional characteristics of microbial communities simultaneously [7]. However, assessment of shotgun data is a difficult job not only because of its data size but also due to its complicated data structure [8]. This is a major impediment to common algorithms.

After the raw reads from metagenomic sequences are generated, the subsequent stage is to evaluate them in order to assess the microbial composition and structure [9]. To achieve this, there are growing numbers of pipelines for bioinformatic assessment [8]. These tools include CLAssifier based on Reduced *K*-mers (CLARK) [10], Genomic Origins Through Taxonomic CHAllenge (GOTTCHA) [11], Metagenomics-Rapid Annotation using Subsystems Technology (MG-RAST) [12], Kraken 2 [13], Quantitative Insights Into Microbial Ecology (QIIME) [14], Metagenomic Phylogenetic Analysis (MetaPhlAn) [15], MOTHUR [16], and metagenomic operational taxonomic units (mOTU) [17]. These pipelines incorporate several algorithms in order to give the greatest possible analysis options. As a result, they entail extensive bioinformatic knowledge and computational infrastructure which may not be available to users of these analytical procedures. Additionally, individual pipelines propose their own protocols, suggested analytical steps, and reference databases. Thus, without an evaluation protocol, selecting a pipeline thru a specified criterion for a particular function can easily become a daunting job.

Bioinformatics pipelines can be categorized into various groups based on several criteria, for example, (1) based on their usage and (2) based on the bioinformatic techniques they use. Based on their usage, these tools can be grouped into (i) self-contained analysis pipelines, i.e., those that integrate various procedures for quality control, sequence clustering, taxonomy assignment, computing diversity measures, and visualizing results and (ii) those that can only be used for a particular step/s in the analysis pipeline [18]. Considering the bioinformatic techniques used, the tools can be grouped into (i) clustering-first approach algorithms, e.g., QIIME, MOTHUR, and MetaPhlAn mOTU, and (ii) assignment-first approach programs, e.g., CLARK, GOTTCHA, Kraken 2, and MG-RAST. Clustering-first methods, also known as alignment-based methods, begin with an OTU-clustering phase in which sequence reads are collected into OTUs founded on their similarity. A representative sequence is obtained from each cluster and then matched, using a homology search tool, to each sequence of the reference database. Lastly, by checking best alignments, the representative sequence and OTU of which it belongs are allocated to a taxonomic group. The assignment-first approaches, however, first compared all reads to a database, then assign the lowest possible taxonomy to any reads or a lower common ancestor (LCA) for a group of sequences of the same taxonomy within the reference database. Then, based on their annotations, the reads are categorized into distinct taxonomic units [9, 11]. Of importance is the fact that clustering-first approaches require a high amount of computing resources. As such, they are almost exclusively the most applied in targeted metagenomics since the data from this approach is greatly reduced. On the other hand, given the complexity of the whole genome shotgun sequence data, assignment-first approaches are recommended since they are not resource intensive as the clustering-first approaches.

Many previous studies using the available tools for shotgun metagenomics have focused on showing how a single analytical step (e.g., sequence pre-processing, OTU clustering or taxonomic assignment) impacted on the microbial classification in real or simulated datasets [9]. In addition, limited literature evaluates the usability and functions of these tools, which often makes the choice of which technique to use unclear [18]. A study to benchmark the most widely used tools for metagenomic analysis showed that the tools most frequently used were not inherently the most precise and that the most effective tool were not automatically the most time-consuming and there was a high level of variation between the available pipelines [8]. Similarly, a study by [19] compared the taxonomic and diversity profiles created by MG-RAST and QIIME using human gut microbiome samples. No statistically significant differences in assignments or alpha diversity measures were found in the study; however, there was a significant difference in beta diversity measures between the two pipelines. The researchers also noted that the more accurate assignments were produced by QIIME, primarily due to the high number of reads that MG-RAST could not classify. In contrast, few studies have been undertaken to assess the methodologies available to comprehensively classify the microbiome within the gastrointestinal tract (GIT) of cattle. This may be attributed to the complexity of the microbial communities that consist of archaea, bacteria, fungi, and protozoa [20]. For example, a previous research by [21] compared taxonomic compositions of rumen microbial communities using Kraken 2 and an in-house pipeline developed based on MOTHUR to compare the rumen fluid RNA collected from cattle with different feed conversion ratios (FCR). The study found out that a similar distribution of the most abundant taxa was found in both pipelines at the phylum level; however, unlike raken 2, MOTHUR was unable to assign sequences to the species level while Kraken 2's ability to identify microbes was restricted due to an absence of some rumen microbiome reference genomes.

In this study, we used fecal microbial sequence data obtained from thirty-six Tanzanian small holder dairy cattle to put forward a comparative analysis of the outcomes of two commonly used assignment-first pipelines, MG-RAST [12] and Kraken 2 [13], with emphasis on the phylum, genus, and species taxonomic assignments. Functionality and usability of the two pipelines were compared and reviewed. Despite their distinct workflows, these two pipelines have been chosen for evaluation because they are the most frequently used and cited pipelines for analyzing metagenomic data. Additionally, no studies have been carried out to compare these pipelines using shotgun data. This research can be of particular use and relevance to scientists who are new to the field or who have limited bioinformatic knowledge to decide which techniques to use in their metagenomics research.

## 2. Materials and Methods

### 2.1. Fecal Sample Collection, DNA Extraction, Library Construction, and Sequencing

Fecal samples were collected purposefully from thirty-six (36), adult cross-breed dairy cattle from Lushoto and Rungwe districts in Tanzania. Lushoto district lies between latitudes 4° and 6°S and longitudes 38° to 39°E in Tanga Region [22], while the Rungwe district is located in Mbeya Region and lies between latitudes 9° 00 and 9° 30 E and longitudes 33°E and 34°S [23]. A clean palpation sleeve and sterile lubricant was used to collect about 250 g of individual fecal samples from the rectum of each cattle and a subsample transferred into a sterile 50 ml falcon tube. Samples were then shipped on ice to the Biosciences east and central Africa (BecA-ILRI) Hub, at the International Livestock Research Institute laboratory, where they were stored at -20°C until further processing.

Fecal DNA was extracted with the QIAamp DNA Stool Mini Kit (Qiagen, USA) according to the manufacturer's instructions using approximately 0.25 g of each fecal sample. Additionally, 2 *μ*l of RNAse A was added during the extraction procedure. The yield and integrity of DNA were determined using a NanoDrop® ND-2000 UV spectrophotometer (Nano-Drop Technologies, Wilmington, DE) and a Qubit 2.0 fluorimeter (Invitrogen, Carlsbad, CA, United States). Sequencing libraries were prepared using the Nextera XT Index Kit (Illumina), following the manufacturer's guidelines. The quality of libraries was assessed using the Agilent 2200 TapeStation (Agilent Technologies, Santa Clara, CA, United States) and a Qubit 2.0 fluorimeter (Invitrogen, Carlsbad, CA, United States). Finally, the libraries were paired-end (2 × 200 bp) sequenced using an Illumina MiSeq v3 (Illumina) System at the BecA-ILRI Hub.

### 2.2. Bioinformatics and Statistical Analysis

#### 2.2.1. Kraken 2

**Figure 1:**
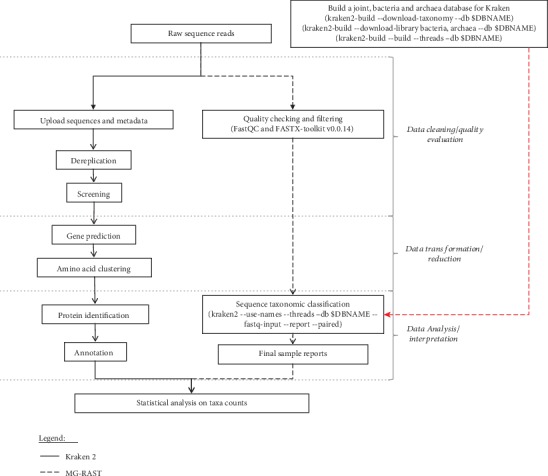
Overview of the workflow used (Kraken 2 and MG-RAST) presenting software parameters used to analyze the data. MG-RAST has two additional steps for data transformation and reduction.

Prior to sequence analysis, filters were used to extract low-quality reads from all samples. Evaluation of sequence quality was done using FastQC software version 0.11.5 (http://www.bioinformatics.babraham.ac.uk/projects/fastqc/). Reads with an average quality score < 20 were then truncated using FASTX-trimmer, a module within the FASTX-toolkit version 0.0.14 (http://hannonlab.cshl.edu/fastx_toolkit/). Following quality control steps, detection of taxa by the *k*-mer approach was done using Kraken 2 [13]. In this pipeline, we used a custom database that had been built in the ILRI Research Computing cluster (http://hpc.ilri.cgiar.org/), for Kraken 2 using RefSeq (version 88) complete bacteria (15,947 genomes), and Archaea (311) genome sequences. To build this joint database, the script kraken2-build was used, with default parameters, to set the lowest common ancestors (LCAs). Microbial classification of each pair of sequences was then done on the basis of their annotations at the lowest taxonomic level by Kraken 2 in the customized standard database. In this operation, Kraken 2's *k*-mer paths allocated each node a specific weight while improving the sensitivity of the classification of species [13]. The --use-names and --report options provided full taxonomic names associated with each classified sequence and standard ranks for each taxon (Figure 1). The complete Kraken 2 database took 4 h 2 m 9.769 s to build on a server with 15 CPUs (2.7 GHz) and 116 GB of RAM, while each sequencing dataset used 35 GB RAM for classification.

#### 2.2.2. MG-RAST

Raw reads were uploaded to MG-RAST for sequence analysis. The analysis options used in the study were (i) removal of artificially replicated sequences produced by sequencing artifacts [24], (ii) using B. taurus, UMD v3.0 database to get rid of any host specific species sequences by means of DNA level matching with Bowtie 2 [25], and (iii) removal of low-quality sequences using a modified DynamicTrim [26]. During trimming, the lowest phred score that was counted as a high-quality base was 15 and sequences were trimmed to contain at most 5 bases below the abovespecified quality. Sequences were then given a taxonomic classification using BLAT [27] and the M5NR database [28]. The taxonomic analysis was done with the MG-RAST default setting: 60% sequence similarity of 15 amino acids and a maximum *e* value cut-off of 1 × 10 − 5. Reads that did not attain the threshold at the chosen taxonomic level were categorized as “Unclassified”, while sequences not assigned to any taxonomic unit fell in the category called “No Hits”. After taxonomic assignment, MG-RAST created a web page to view, analyze, and download results so that they can be used for comparison with other tools [29] (Figure 1).

#### 2.2.3. Statistical Analysis

To assess the taxonomic assignment power of the two algorithms, we extracted the outcomes acquired at the phylum, genus, and species levels. Paleontological STatistics software package for education and data analysis tool (PAST v3.13, 30) was used to calculate diversity measures. Alpha diversity indices calculation included Chao1 minimal richness index [30], inverse Simpson diversity index [31, 32], and Shannon diversity index [33] . We used a *t*-test to assess for statistically significant differences between each index and relative abundance of the various taxa assigned by the two tools.

## 3. Results

Two bioinformatic methods, Kraken 2 and MG-RAST, were used in this research to acquire taxonomic classifications (bacteria and archaea) of Tanzanian dairy cattle's feces. For most analytical steps, the two tools had a related basic algorithm (Figure 1). However, significant differences in taxonomic assessment, metagenomic function assignment, and visualization were noted.  Table 1 provides an overview of the characteristics and functionality of the two tools.

### 3.1. MG-RAST and Kraken 2's Taxonomic Distribution of Microbial Profiles

Taking into account the complete amount of microbial species in the specimens, Kraken 2 recognized, in all taxonomic ranks, a greater amount of bacterial and archaeal phylotypes than MG-RAST (Table 2). At the phylum level, bacterial profile findings disclosed a comparable taxon distribution among the four most common species categorized by both pipelines (Tables 2 and 3), with Proteobacteria, Firmicutes, Bacteroidetes, and Actinobacteria being most abundant and responsible for about 80% of the total microbial population. However, Bacteroidetes was detected in lower abundance by Kraken 2 (9.7%), than by MG-RAST (12.7%), while Actinobacteria had higher abundance (2.9%) in Kraken 2 than MG-RAST (1.25%). Nevertheless, these variations were not regarded as statistically significant. In total, both pipelines detected 40 bacterial phyla. Of these phyla, 26 were identified by both pipelines, 12 were solely identified by Kraken 2 while MG-RAST exclusively identified two (Table 2). The sequences assigned to the Bacteria root, but with no taxonomical assignment at the phylum level, were reported as “Unclassified”; those sequences were on average 0.04% for MG-RAST and 0% for Kraken; this difference was statistically significant between the two tools (Supplementary Table (available here)).

Across the two pipelines, a total of 1289 different genera were detected. Although the two pipelines had some resemblance (497 genera commonly detected), Kraken 2 exclusively identified an extra 694 genera while MG-RAST solely identified 98 genera (Table 2). The two pipelines identified Pseudomonas as the most abundant genus: Kraken 2 32.64% and MG-RAST 32.42%. There were significant variations among the most abundant taxa on the genus level (relative abundance > 1%) in the two pipelines. Two genera *Comamonas* (*P* < 0.001) and *Acinetobacter* (*P* = 0.01) were identified in higher abundance by Kraken 2 while *Bacteroides* (*P* = 0.03), *Acidovorax* (*P* < 0.001), and *Clostridium* (*P* < 0.001) had higher abundances in MG-RAST.  Table 3 presents an overview of the top genera detected by both pipelines. Kraken 2 and MG-RAST detected 4465 and 1481 species, respectively (Table 2). Notable differences in the six most abundant species identified by the two algorithms were the higher abundance of *Prevotella ruminicola* and *Escherichia coli* in Kraken 2 whereas *Pseudomonas fluorescens, Comamonas testosterone*, *Pseudomonas putida*, and *Pseudomonas stutzeri* were more abundant in MG-RAST. A full list of phylotypes (in all taxonomic ranks) recognized across the two pipelines is provided in Supplementary Table.

In terms of archaeon identification, both methods identified five phyla, with four being identified by both methods. In addition, significant differences were observed in the two methods at the genus and species levels. Kraken 2 generated 105 and 236 genera and species, respectively, while MG-RAST detected 60 genera and 88 species. 54 genera and 72 species were commonly detected by the two methods (Table 1). Individual algorithm differences in archaea identification can be found in Table 4 and Supplementary Table.

### 3.2. Taxon-Related Abundance Differences between Lushoto and Rungwe Samples

**Figure 2:**
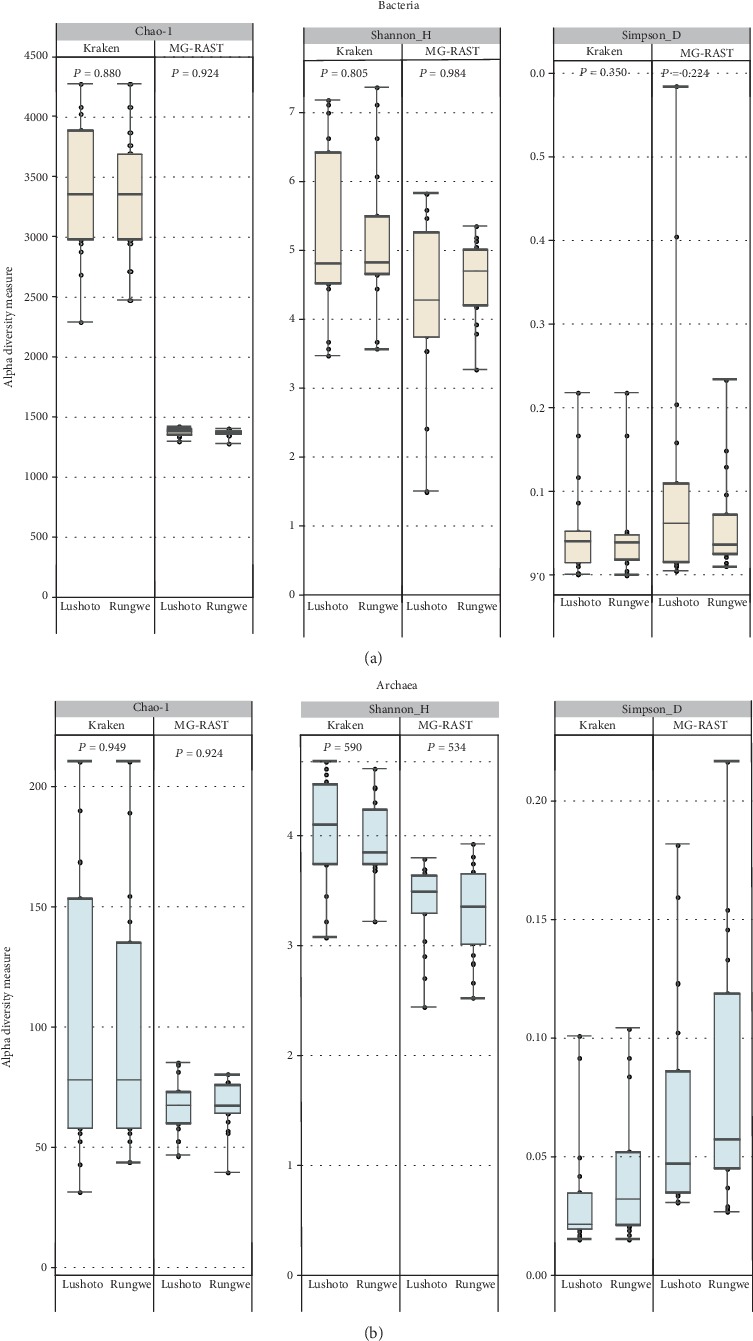
Alpha diversity matrices of bacteria (a) and archaea (b), between Lushoto and Rungwe samples.

To assess how the two approaches affected biological interpretation of bacteria and archaeon composition and community structure, comparisons of fecal microbiota were made between Lushoto and Rungwe samples. Microbial abundance differences at phylum and genus levels between Lushoto and Rungwe datasets were observed to be minimal (<1% of all microbial population), irrespective of the pipeline (Table 2 and Supplementary Table). In this regard, within bacteria, one genus *Planococcus* and two genera *Hafnia* and *Spiroplasma* were detected in Lushoto and Rungwe datasets, respectively, with MG-RAST classification. This was contrary to Kraken 2 that identified these genera in the two regions. At the species level, 22 and 18 species were identified only in Lushoto and Rungwe samples, respectively, based on Kraken 2 while MG-RAST detected 12 samples exclusively in Lushoto and 9 samples only in Rungwe. Within archaea, only one difference, *Methanosarcina* sp. *WH1*, was observed between the two regions when samples were classified using Kraken 2. This species was only detected in Rungwe samples. Assessment of the differences in microbial abundance between Lushoto and Rungwe datasets using an independent *t*-test revealed no significant difference in all microbial taxa detected in both regions. In addition, alpha diversity indexes (Shannon, Simpson, and Chao 1), of bacteria and archaea, at the species level, indicated no significant difference when they were compared between Lushoto and Rungwe groups within the two pipelines (Figure 2).

### 3.3. Usability and Overall Performance

Each pipeline provides avenue for analysis of shotgun metagenomic sequencing data. There are, however, major variations in the development of each pipeline. MG-RAST offers an interactive service where the researcher uploads information to a web application and chooses a number of parameters for quality control. The data then passes through several analytical steps automatically, and then, the user is left to produce abundance profiles, functional features, and visualizations. Moreover, MG-RAST analysis is conducted using a web-based graphical user interface (GUI) making it readily available to all researchers with an internet connection. In addition, to process multiple samples, it does not need to be installed or does it require a powerful computer. Furthermore, MG-RAST acts as a public database for metagenomic shotgun datasets and as such, investigators can compare and investigate other publicly available datasets. Navigation around the website is easy, and the options for analyzing data are clear and well-explained. In contrary, analysis with MG-RAST is very time-consuming as the outputs require a lot of cleaning because of the multiple read annotations. Although it is not hard to do, data cleaning is time-consuming and would be hard to finish in a timely way for big datasets. Besides, while in Kraken 2 the analysis can start immediately, the samples must go through a quality control in MG-RAST before they can be analyzed. MG-RAST gives a precedence to data submitted for analysis based on when the dataset will be publicly released, and the wait for private data to endure quality control can take up to several weeks.

Kraken 2 on the other hand is a command-based algorithm where the user uses a set of sequential scripts to achieve classification in a custom or default database. The main challenge in Kraken 2 is that this algorithm may be tasking especially if the user has to build their own custom database using genomes found in the RefSeq. Because of its quick analysis time, Kraken 2 is more likely to be used to analyze a large dataset. Moreover, researchers with command line competence and looking to carry out complex analysis may prefer Kraken 2 due to its increased user freedom. The Kraken 2 pipeline was adapted to include all reference genomes in RefSeq, which has led to more bacterial species and phylotypes being identified. However, the findings of the classification of archaea and some of the bacterial species recognized by Kraken 2 should be assessed judiciously as many phylotypes detected have not yet been defined in the rumen. Notwithstanding our strategy, the only way to improve is to strengthen databases continuously through the inclusion of extra data on whole-genome sequences of rumen isolates and single-cell sequencing of noncultured rumen microbes since rumen microorganisms are still limited in their capability for culture.

In addition to ease of usage, runtime and memory requirements for shotgun metagenomic algorithms are important factors to consider and should not be underestimated. The run time varied between the two tools with Kraken 2 using 4 hours while MG-RAST took 2 days on average. The runtime of a user with MG-RAST can strongly rely on a number of variables, including present load, software upgrades, and work priorities. At the peak memory usage, Kraken 2 used 35 GB RAM per sample. We were unable to carry out this assessment on MG-RAST as it was only accessible through a website.

## 4. Discussion

In this research, the taxonomic results of two metagenomic assessment pipelines, Kraken 2 and MG-RAST, are compared using fecal metagenome data of dairy cattle reared by smallholder farmers in Tanzania. The emergence of high-throughput sequencing has significantly improved our understanding about the ecology and functional ability of different ecosystems including the gastrointestinal tract (GIT) of cattle. However, the functional results and the biological interpretation of this information rely heavily on the computational methods used [1, 9]. We observed that while there is little variation between the two pipelines in terms of taxonomic classifications and diversity measures, there were substantial usability differences, particularly in time taken for analysis of samples and the ease of use.

### 4.1. Comparisons of Microbial Composition and Abundance

In this research, both analysis tools showed that the feces of cattle were dominated by Proteobacteria followed by Bacteroidetes and Firmicutes. The dominance of Proteobacteria in these samples, without any health or production effect, is of particular interest given that earlier researchers have found a mechanistic interplay between Proteobacteria, intestinal immune response, and inflammation [34]. A recent publication of nursing calves from 5 beef farms with greater concentrations of Proteobacteria comparable to this research by [35] proposed that greater concentrations of Proteobacteria could have been a farm-associated effect, possibly from management practices. Specifically, the foregoing authors noted that Proteobacteria-enriched microbiota was observed in farms that had the highest antimicrobial treatment rates leading to their speculation that practices of antimicrobial use could have a wider or cumulative impact on farms in which their recurrent uses result in development, regardless of individual antimicrobial exposures, of a specific microbiota in farm animals. In this study, we were unable to confirm this assertion since the sampled farms had no proper recording systems on the use of antimicrobials. However, some studies, although in humans, have corroborated this theory [33, 34].

Although Bacteroidetes abundance was higher in MG-RAST than Kraken 2, this difference was not significantly different. This phylum contains a wide range of individuals who can be found in several ecosystems including the mammalian and insect guts, soil, and both fresh and salt water ecosystems [35–37]. A typical characteristic of ecological Bacteroidetes is their capacity to break down complex glycans, for example, agarose, alginate, cellulose, chitin, and hemicellulose [38]. It has also been observed that Bacteroidetes are involved in the spread of antimicrobial resistance genes through horizontal gene transfer [39]. Firmicutes were the second most abundant taxonomic group. This phylum is believed to play a crucial part in the harvest of energy [40]. Moreover, members within this phylum can have both positive and negative influences on the host animal. Some species within this phylum, for instance, engage in the degradation of complex organic materials such as cellulose, chitin, xylan lignocellulose, and xylose and even act as useful probiotics and nitrogen fixing agents [41]. Conversely, several species are potentially hazardous species that cause several diseases in animals and humans [42].

Interestingly, both methods had an underrepresentation of potentially important Cyanobacteria phyla (Supplementary Table), supporting findings from prior research of low abundance of these phototrophic oxygenic bacteria in dairy and beef cattle rumen [40, 41]. Cyanobacteria can be both heterocystous and nonheterocystous [43]. Although the ruminal environment is commonly deemed anaerobic, significant levels of oxygen in the rumen fluid can be identified [44], suggesting that the occurrence of cyanobacteria in the rumen may be associated with the scavenging of oxygen and the fermentation of sugar under restricted aerobic environments [21]. Whereas Cyanobacteria has been extensively detected in aqueous and soil environments [45, 46], it is important to point out that the identification of this phylum in the gut of humans raises grave questions regarding their role in aphotic and anaerobic habitats like the rumen [47]. Recent investigations have shown gut Cyanobacteria to be very conserved, but their 16S rRNA phylogenetic tree was different from the photosynthetic ones; this has led to the designation of a new putative class called Melainabacteria [47], whose members were able to ferment a variety of sugars in the gut [48]. Similar to other previous studies [21, 39] neither Kraken 2 nor MG-RAST identified Melainabacteria in the samples, showing the need for further research to distinguish their role in the gut of cattle.

The three most dominant genera in both pipelines were *Pseudomonas*, *Comamonas*, and *Acinetobacter*. These genera are not only important in the rumen ecosystem but have also been linked with other environmental roles. For instance, there are many reports indicating that Pseudomonas spp. produced antifungal compounds, siderophores, and indole acetic acid (IAA). However, they are considered powerful human pathogens that may cause respiratory, urinary, and gastrointestinal tract infections [49]. Although low in abundance, *Acinetobacter* and species within it are common in nature and some strains are known to be engaged in biodegradation of a variety of pollutants. They are also involved in the manufacture of products such as lipases, proteases, cyanophine, bioemulsifiers, and various types of biopolymers [42]. Furthermore, Acinetobacter has been reported to have a role in phosphate solubilization and nitrogen fixation [50].

In order to fully comprehend the role of the rumen microbiota, it is vital to define organisms at the level of the species since distinct species can have distinct tasks and niches within the same genus. Unlike MG-RAST that used an already preformed database, the Kraken 2-based approach used a custom reference database assembled based on all identified microbial genomes, at that time. As a result, higher microbiota resolution was generated by Kraken 2, enabling the program to uniquely identify 3550 species compared to MG-RAST that identified 557 species. However, Kraken 2 is also limited by the lack of all reference genomes for rumen microorganisms. For instance, identification of *Xenorhabdus doucetiae*, a soil bacterium, had not been earlier recoded in the rumen contents' metagenome [51]. Identification of this bacterial species may show that Kraken 2 did not correctly identify the microbe, since the reference genome data was based mostly on all microbial genomes annotated in the NCBI database. These organisms may have been identified in the rumen, however, since cattle can eat soil, which makes it possible to detect them temporarily [21].

The Archaea domain was dominated by the phylum Euryarchaeota in both pipelines. However, at the genus level, *Hyperthermus* and *Methanobrevibacter* were identified as the most predominant genus by Kraken 2 and MG-RAST, respectively (Supplementary Table). Previous studies have reported that *Methanobrevibacter* was the most abundant archaeal population in the rumen based on DNA datasets [48, 49]. However, further studies are needed to determine whether the differences in archaeal abundance between these two pipelines have a methodological influence or are controlled by diet, host animal, or management strategies. The differences in the two algorithms were further shown by contrasting results in species identified. For example, Kraken 2 was able to detect *Candidatus Methanoplasma termitum* and *Candidatus Methanomethylophilus alvus*, which were not identified by MG-RAST pipeline. A similar finding was found in a previous study by [21]. These two species encode pathways required for hydrogen-dependent methylotrophic methanogenesis by reduction of methyl substrates, without the ability to oxidize methyl substrates to carbon dioxide [52]. Thus, it is possible that these microbes reside in the rumen. Further, Kraken 2 uniquely identified *Methanogenic archaeon ISO4-H5*, a member of the order Methanomassiliicoccales, that had been previously shown to exhibit a genome size of 1.9 Mb and GC content of 54%, similar to *Candidatus Methanoplasma termitum* and *Candidatus Methanomethylophilus alvus* [53]. Given the low relative abundance and the species not being identified by both pipelines, future analysis with databases enriched with sequences from *Methanogenic archaeon ISO4-H5* as well as its isolation, culture, and characterization may provide further evidence of this possibility.

### 4.2. Functionality and Usability Comparisons

MG-RAST analysis is influenced by the server times and its data uploading limits. The user submits raw data specifying if the data will be private or public. The choice of when the data will be made public then assigns what priority MG-RAST provides to this data. Data that is meant to remain private is usually assigned the lowest priority option. Therefore, the time needed to finish the analysis by the MG-RAST server is based on the selected priority level and the number of jobs submitted in the analysis queue by all MG-RAST users. Conversely, Kraken 2 is entirely installable and users can begin their assessment as immediately after installation is finished. Nevertheless, installation needs some basic bioinformatic expertise, particularly when building a custom database. The time needed to complete the analysis in Kraken 2 depends on a number of factors, in particular the amount of data and the bioinformatics skills of the user. Kraken 2 is by far the quicker pipeline due to the fact that the database is not preloaded into memory by default. Such preloading with a RAM Disk is possible and reduces the execution time of Kraken 2 but requires RAM space at least equal to the database size. When using the full RefSeq database, this tradeoff should be regarded, which could significantly increase runtime. MG-RAST and other webservers do not require high computing resources, but even so require a decent and steady internet bandwidth, and the users rely on external computer resources that they have no command over.

Similar to previous studies, [18, 19], MG-RAST provided a lower level of accuracy as a higher number of reads were assigned to the “Unclassified” category. This is not surprising given the optimization of MG-RAST to evaluate short-read, low-error data. However, MG-RAST has some outstanding utilities. For example, following the search in the reference database, MG-RAST produces a multi-FASTA file, for each sample, which contains all reads that were assigned an identity. This file is easily accessible and extremely useful for resolution of multiple annotations generated by MG-RAST, examining unclassified reads, and also picking specific reads to perform downstream analyses such as multiple sequence alignments.

There are limitations to this study. We recognize that there are some differences between the analytical methods used by the two pipelines that can affect comparability. For instance, the quality control parameters for MG-RAST differs from those used in Kraken 2, and we were unable to determine the SILVA database version used for MG-RAST taxonomy assignment. This study illustrates how bioinformatics pipeline selection can affect metagenomic sequencing data analysis. The strength of this study is that it used a larger dataset. However, given that the data used in this study are all the same type of sample and came from one project, it should be emphasized that the taxonomic composition variations in the samples could be from the differences in the pipelines.

## 5. Conclusions

There are often many algorithms, software packages, or pipelines in the field of bioinformatics that can be used to conduct a single job. Even for skilled bioinformaticians, it is not simple to choose a single “best tool”. In this study, we were able to carry out a comparative metagenomic assessment of cattle fecal microbial composition using both Kraken 2 and MG-RAST algorithms. Our results indicated that Kraken 2 and MG-RAST had comparable results in terms of the phylum detected and that regardless of which pipeline selected, you are likely to generate a reliable overview of sample composition. However, we observed that Kraken 2 generated a more accurate taxonomic identification given the higher number of “Unclassified” reads in MG-RAST. Nevertheless, less experienced users may find MG-RAST simpler than Kraken 2. Therefore, we propose that MG-RAST could be useful for first-time users to acquaint themselves with the analysis and output of metagenomic analysis.

## Figures and Tables

**Table 1:** Comparison of the functionality and features of MG-RAST and Kraken 2.

	Kraken 2	MG-RAST
License	Open-source	Open-source
Implemented in	C++ and Perl	Perl
Current version (at 23.05.19)	v2.0.8-beta	4.0.3
Website	http://ccb.jhu.edu/software/kraken/ (Wood & Salzberg 2014)	https://www.mg-rast.org/ (Meyer et al. 2008)
Web-based interface	No	Yes (at website above)
Primary usage	Command line	GUI (at website above)
Sequencing technology compatibility	Illumina, 454, Sanger, Ion Torrent, PacBio	Illumina, 454, Sanger, Ion Torrent, PacBio
Quality control	No	Yes
Taxonomic analysis/assignment	*k*-mers	BLAT
Taxonomy	Yes	Yes (E)
Function	No	Yes
Fastq	Yes	Yes
Zipped	Yes	Yes
Paired	Yes	Yes (R)
Diversity analysis	NO	Alpha
Phylogenetic tree	NO	YES
Visualization	NO	PCA plots, heat maps, pie charts, bar plots, krona and Circos for visualisation

“(E)” indicates if the tool infers Eukaryotic taxa and/or functional analysis. GUI means graphical user interface, and “(R)” means the server recognizes paired-end data but seems to treat reads separately. Part of this figure was adapted from the pipeline published by [18].

**Table 2:** Evaluation of taxonomic phylotypes by each technique.

Phylotypes	Kraken 2		MG-RAST		Commonly detected phylotypes
	Lushoto (no.)	Rungwe (no.)	Lushoto (no.)	Rungwe (no.)	
Bacteria					
Phyla	38	38	28	28	26
Genera	1191	1191	595	596	497
Species	4462	4465	1479	1481	933
Archaea					
Phyla	5	5	5	5	4
Genera	105	105	60	60	54
Species	235	236	88	88	72

**Table 3:** Most abundant bacteria according to the two classification approaches.

Taxa	Kraken 2	MG-RAST	*P* value
	Mean ± SE (%)	Mean ± SE (%)	
Phyla			
Proteobacteria	75.92 ± 4.1	75.12 ± 3.06	0.88
Firmicutes	9.69 ± 2.2	9.29 ± 1.63	0.88
Bacteroidetes	9.22 ± 1.41	12.7 ± 1.54	0.1
Actinobacteria	2.9 ± 0.41	1.25 ± 0.13	<0.001
Tenericutes	0.72 ± 0.11	0.19 ± 0.05	<0.001
Genus			
*Pseudomonas*	32.64 ± 4.1	32.42 ± 3.29	0.97
*Comamonas*	8.38 ± 1.34	3.57 ± 0.54	<0.001
*Acinetobacter*	6.72 ± 1.82	1.87 ± 0.56	0.01
*Janthinobacterium*	2.2 ± 1.36	1.08 ± 0.22	0.42
*Bacteroides*	1.83 ± 0.4	3.72 ± 0.75	0.03
*Acidovorax*	1.61 ± 0.23	6.18 ± 0.83	<0.001
*Stenotrophomonas*	1.85 ± 0.35	1.75 ± 0.3	0.83
*Clostridium*	1.19 ± 0.26	2.97 ± 0.54	<0.001
Species			
*Pseudomonas fluorescens*	6.77 ± 1.69	21.67 ± 2.81	<0.001
*Comamonas testosterone*	1.03 ± 0.35	3.57 ± 0.54	<0.001
*Pseudomonas putida*	1.34 ± 0.38	2.34 ± 0.18	0.02
*Pseudomonas stutzeri*	1.46 ± 0.49	1.66 ± 0.37	0.74
*Escherichia coli*	1.22 ± 0.21	0.45 ± 0.13	<0.001
*Prevotella ruminicola*	0.83 ± 0.13	0.21 ± 0.05	<0.001

**Table 4:** Most abundant archaea according to the two classification approaches.

Taxa	Kraken 2	MG-RAST	*P* value
	Mean ± SE (%)	Mean ± SE (%)	
Phyla			
Euryarchaeota	90.96 ± 0.41	94.44 ± 0.44	<0.001
Crenarchaeota	6.59 ± 0.27	4.45 ± 0.39	<0.001
Thaumarchaeota	2.3 ± 0.25	0.54 ± 0.06	<0.001
Korarchaeota	0.07 ± 0.03	0.49 ± 0.08	<0.001
Genus			
*Methanocaldococcus*	8.17 ± 1.47	2.4 ± 0.16	<0.001
*Methanosarcina*	7.62 ± 0.51	12.83 ± 0.82	<0.001
*Thermococcus*	6.79 ± 0.51	2.51 ± 0.19	<0.001
*Methanocorpusculum*	3.53 ± 0.59	6.39 ± 0.98	0.01
Species			
*Methanobrevibacter ruminantium*	3.62 ± 0.7	9.51 ± 1.28	<0.001
*Methanococcus maripaludis*	2.82 ± 0.27	3.42 ± 0.23	0.1
*Methanobrevibacter smithii*	1.84 ± 0.27	15.87 ± 1.85	<0.001
*Methanocorpusculum labreanum*	1.97 ± 0.54	6.39 ± 0.98	<0.001
*Methanosarcina barkeri*	1.6 ± 0.19	3.96 ± 0.23	<0.001

## Data Availability

The datasets generated and/or analysed during the current study are available in the https://www.mg-rast.org/linkin.cgi?project=mgp81260.

## Supplementary Materials

Supplementary Materials The supplementary material provided is a full list of OTUs (in all taxonomic ranks) identified by the two pipelines (MG-RAST and Kraken).
